# Antiferroelectric Nature of CH_3_NH_3_PbI_3−x_Cl_x_ Perovskite and Its Implication for Charge Separation in Perovskite Solar Cells

**DOI:** 10.1038/srep30680

**Published:** 2016-07-29

**Authors:** Galhenage A. Sewvandi, Kei Kodera, Hao Ma, Shunsuke Nakanishi, Qi Feng

**Affiliations:** 1 Department of Materials Science and Engineering, Faculty of Engineering, University of Moratuwa, Katubedda, SriLanka; 2 Department of Advanced Materials Science, Faculty of Engineering, Kagawa University, 2217-20 Hayashi-cho, Takamatsu 761-0396, Japan

## Abstract

Perovskite solar cells (PSCs) have been attracted scientific interest due to high performance. Some researchers have suggested anomalous behavior of PSCs to the polarizations due to the ion migration or ferroelectric behavior. Experimental results and theoretical calculations have suggested the possibility of ferroelectricity in organic-inorganic perovskite. However, still no studies have been concretely discarded the ferroelectric nature of perovskite absorbers in PSCs. Hysteresis of P-E (polarization-electric field) loops is an important evidence to confirm the ferroelectricity. In this study, P-E loop measurements, in-depth structural study, analyses of dielectric behavior and the phase transitions of CH_3_NH_3_PbI_3−x_Cl_x_ perovskite were carried out and investigated. The results suggest that CH_3_NH_3_PbI_3−x_Cl_x_ perovskite is in an antiferroelectric phase at room temperature. The antiferroelectric phase can be switched to ferroelectric phase by the poling treatment and exhibits ferroelectric-like hysteresis P-E loops and dielectric behavior around room temperature; namely, the perovskite can generate a ferroelectric polarization under PSCs operating conditions. Furthermore, we also discuss the implications of ferroelectric polarization on PSCs charge separation.

Organic-inorganic perovskite semiconductors have allured massive scientific attention since their incorporation into photovoltaic devices due to soaring efficiencies of PSCs^1^,^2^,^3^,^4^. Anomalous behavior of PSCs urges researchers to investigate the fundamental properties of the perovskites absorbers which presumed to be reasons for these mysterious behaviors. Among the fundamental properties, ferroelectricity and ion migration of the perovskite absorber have been attracted the much interest of scientific community because they can generate a polarization on the interfaces of perovskite/TiO_2_ and perovskite/HTM which will affect the charge transfer mechanism in PSCs^5^,^6^,^7^,^8^,^9^,^10^,^11^,^12^,^13^,^14^,^15^.

Experimental results and theoretical calculations have suggested the possibility of ferroelectricity in CH_3_NH_3_PbI_3_ perovskite^5^,^6^,^7^,^8^,^9^. Ferroelectric domains about 100 nm in a size have been observed by using piezoforce microscopy (PFM) and ferroelectric domain switching has also been achieved by poling^10^. Larger spontaneous polarizations have been seen in larger perovskite crystals with an external electric field and the retention of ferroelectric polarizations has also been observed after removal of the electric field, larger crystals have showed longer retention behavior compared to the smallers^11^. A 180° domain phase switching on the CH_3_NH_3_PbI_3_ thin films has been observed in PSCs^12^. In contrary, some studies have demonstrated the absence of ferroelectricity^13^,^14^,^15^,^16^. Very recently detailed structural studies on CH_3_NH_3_PbI_3_ perovskite using neutron diffraction have revealed the phase transitions from the orthorhombic phase to tetragonal phase at 165 K and the tetragonal phase to cubic phase at 327 K, and also the disordered orientation of CH_3_NH_3_^+^ cation in the tetragonal CH_3_NH_3_PbI_3_ around room temperature, which excludes the possibility of spontaneous polarization by the orderly orientated CH_3_NH_3_^+^ cations in the perovskite around room temperature^17^,^18^,^19^. Monte Carlo simulations have shown the formation of either antiferroelectric or ferroelectric domains in CH_3_NH_3_PbI_3_ perovskite with reducing the temperature^17^,^18^. Nonetheless, significant ambiguities still remain regarding the ferroelectricity because the displacement of positive and negative charge centroids by shifting the position of Pb(II) ion in the PbI_6_ octahedron and the displacement of CH_3_NH_3_^+^ cation can also generate spontaneous polarization in the organic-inorganic perovskites similar to the most ferroelectric metal oxide perovskites. In the present study, P-E loop measurements and in-depth structural study of CH_3_NH_3_PbI_3−x_Cl_x_ perovskite were carried out to clarify the doubtful ferroelectric behavior.

## Results

### Structural study

**Figure 1:**
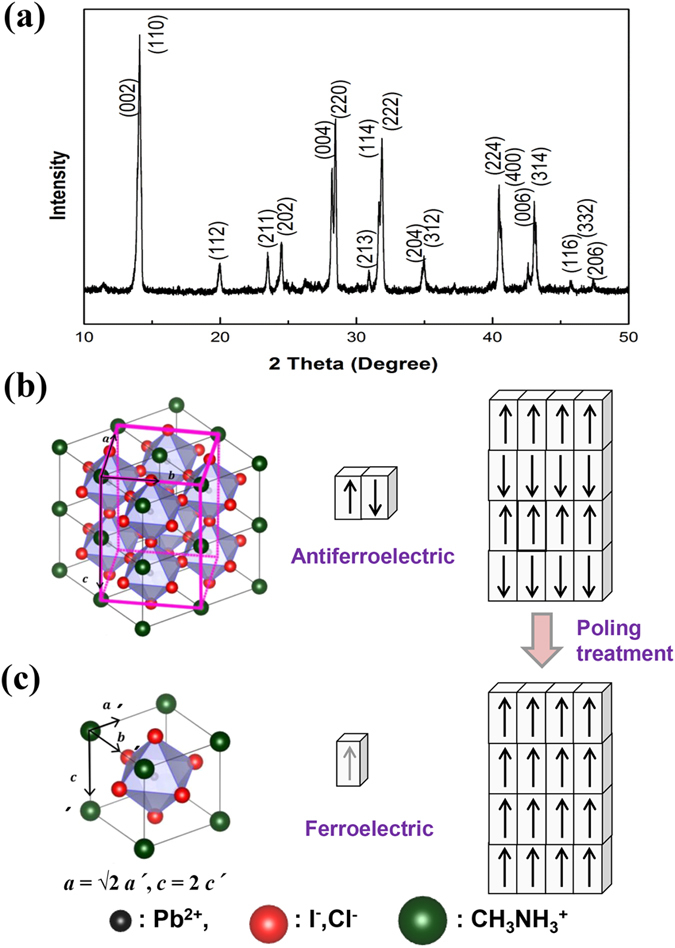
Structural analysis of CH_3_NH_3_PbI_3−x_Cl_x_. (**a**) Powder X-ray diffraction (XRD) pattern of the synthesized CH_3_NH_3_PbI_3−x_Cl_x_. (**b**) Tetragonal I4/mcm superlattice crystal structure of CH_3_NH_3_PbI_3−x_Cl_x_ perovskite. (**c**) Sublattice cell structure of CH_3_NH_3_PbI_3−x_Cl_x_ perovskite.

It is pertinent to evaluate lattice parameters to analyze ferroelectric behavior because ferroelectric behavior of the well-known ferroelectric metal oxide perovskites, such as BaTiO_3_ and Pb(Zr, Ti)O_3_, depends strongly on their crystal parameters. The crystal structural parameters of the synthesized CH_3_NH_3_PbI_3−x_Cl_x_ were measured using powder X-ray diffraction (XRD). The all diffraction peaks in the XRD pattern (Fig. 1a) can be indexed into the I4/mcm space group of tetragonal lattice parameters ***a*** = 8.87405 and ***c*** = 12.6380 Å, a superlattice structure (Fig. 1b) consists of tetragonal sublattice cells with lattice constants ***a′*** = 6.27490 ( = ***a***/√2) and ***c ′*** = 6.31899 Å ( = ***c***/2) (Fig. 1c). The ***c ′***/***a′*** ratio (1.007) of the CH_3_NH_3_PbI_3−x_Cl_x_ perovskite is greater than 1. And that value of ferroelectric tetragonal BaTiO_3_ is 1.010^20^. Neutron diffraction study has shown a decrease in the ***c ′***/***a ′*** ratio of the tetragonal CH_3_NH_3_PbI_3_ perovskite towards unity at 327 K, the phase transition temperature from tetragonal to cubic structure^18^.

### P-E hysteresis loops

**Figure 2:**
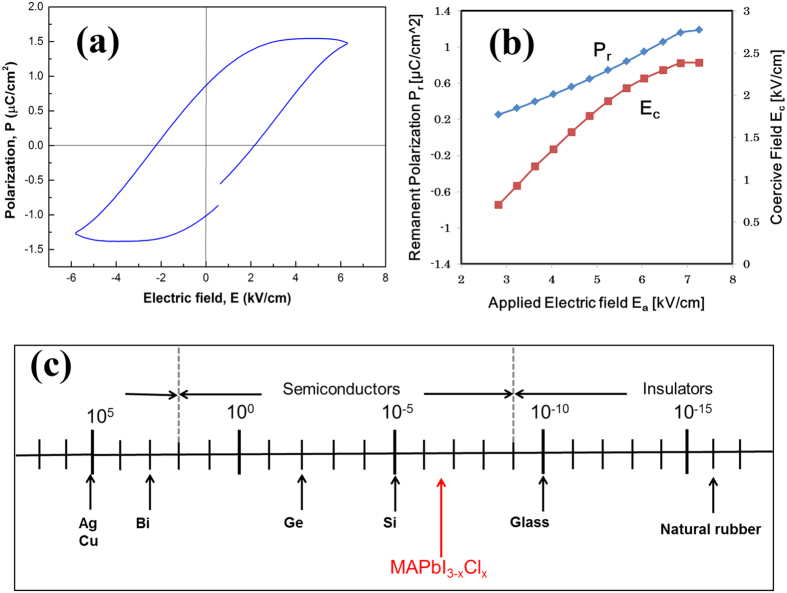
Ferroelectric and semiconducting behavior of CH_3_NH_3_PbI_3−x_Cl_x_. (**a**) Polarization-electric field (P-E) hysteresis loops of ferroelectric CH_3_NH_3_PbI_3−x_Cl_x_ perovskite measured at room temperature and 2 kHz. (**b**) Changes of remanent polarization and coercive field with applied electric field for CH_3_NH_3_PbI_3−x_Cl_x_. (**c**) Systematic representation of conductivity of CH_3_NH_3_PbI_3−x_Cl_x_.

The ferroelectric hysteresis loop is the best experimental proof for the analysis of ferroelectric nature of materials. The ferroelectrics of CH_3_NH_3_PbI_3−x_Cl_x_ perovskite can be confirmed from its P-E hysteresis loops (Fig. 2a). It shows a remnant polarization, P_r_, of about 1.0 μC/cm^2^ and a coercive field, E_c_, of about 2.2 kV/cm after applying an electric field of 6 kV/cm. Although the I4/mcm space group of CH_3_NH_3_PbI_3−x_Cl_x_ perovskite is centrosymmetric, a nonpolar phase, it has been reported that some perovskites with I4/mcm space group, such as SrTiO_3_ and SrTaO_2_N, show ferroelectric behavior due to their antiferroelectrics and local dipoles^21^,^22^,^23^,^24^. Such perovskites show a complex ferroelectric behavior. The nonpolar antiferroelectric phases, for example AgNbO_3_ and NaNbO_3_, can be switched to ferroelectric phases after poling treatment, and these perovskites are used as ferroelectric materials^25^,^26^. This phenomenon in relation to CH_3_NH_3_PbI_3−x_Cl_x_ perovskite is depicted in Fig. 1bc.

We estimated the remanent polarization (P_r_) and the coercive field (E_c_) values from the P-E loops measured at different applied electric field intensities (E_a_) to find a relationship between the applied field and polarization which can correlate to the PSCs I-V measurement conditions. It should be noted that polarization can also arise from conductive effects because our measurements were performed at a high frequency of 2 kHz, the polarization by the movement of ions approaches to zero due to their low mobility (10^−9^ cm^2^ V^−1^ s^−1^ for I^−^)^27^. Some theoretical calculations have suggested polarization effect in perovskite solar cells to electron trappings at the interfaces of TiO_2_/perovskite and perovskite/HTM^28^. The electron trappings at the interfaces can be attributed to low conductivities of TiO_2_ and HTM compared to the perovskite; namely, low conductivities at TiO_2_/perovskite and perovskite/HTM interfaces. In the present study, to remove the electron trapping we have used perovskite pellet samples with Au-electrodes on the both sides for the P-E hysteresis measurement. Therefore a large electrons trapping cannot be expected at the Au/Perovskite interface due to the high conductivity of Au. There may be some trapping electrons at the interface of our pellet. However, it could not generate a large polarization like the normal ferroelectric materials in the order of μC/cm^2^. We think the polarization from the electron trapping must be less than in an order of nC/cm^2^ in our measurements. Therefore, we believe our results represent mainly ferroelectric polarization. The P_r_ and E_c_ gradually increase with increasing the E_a_ to about 7 kV/cm and then reach saturation (Fig. 2b). At the open-circuit conditions of PSCs, assuming the thickness of absorber layer = 300 nm and the cell open-circuit potential (V_oc_) = 0.9 V^29^, the photo-induced internal-electric field will reach to 30 kV/cm. The result of Fig. 2b suggests that it can switch the antiferroelectric absorber layer to its ferroelectric phase; thus, polarize the absorber layer by the photo-induced internal-electric field. Namely, the antiferroelectric perovskite absorber layer can be polarized by itself generated photo-induced internal-electric field and we would like to call it as a self-poling effect. The switching antiferroelectric phase to the ferroelectric phase should be considerably easy because the Curie temperature (T_c_) of CH_3_NH_3_PbI_3−x_Cl_x_ perovskite, 51 °C (see next section), is close to the room temperature (poling treatment temperature).

A conducting measurement revealed that CH_3_NH_3_PbI_3−x_Cl_x_ perovskite sample has a conductivity of 6.47 × 10^−7^ S/cm locating in the range of semiconductors, as shown in Fig. 2c where conductivities of few typical materials varying from semiconductors to insulators are also depicted. This result indicates that CH_3_NH_3_PbI_3−x_Cl_x_ perovskite is an antiferroelectric semiconductor material and can be switched to a ferroelectric semiconductor after poling treatment; namely, it is a semiconductor with a spontaneous polarization, different from the typical ferroelectric materials which are insulators with spontaneous polarizations^30^.

### Phase transition

**Figure 3:**
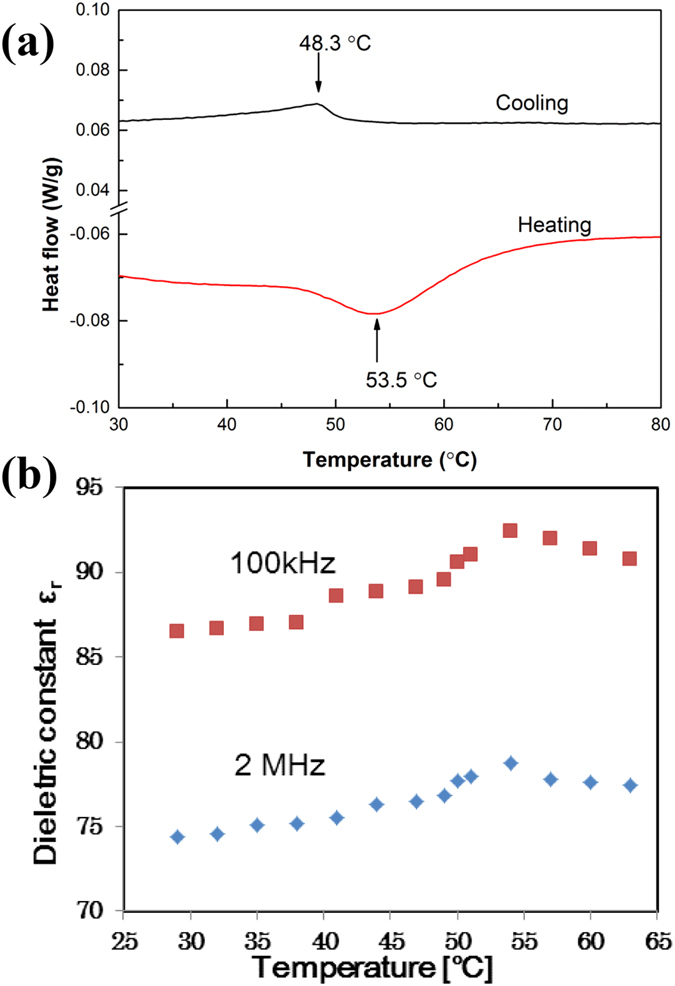
Evidences for the phase transitions. (**a**) DSC plots of CH_3_NH_3_PbI_3−x_Cl_x_ perovskite. (**b**) Temperature dependences of the dielectric constant of CH_3_NH_3_PbI_3−x_Cl_x_ perovskite measured at 2 MHz and 100 kHz, respectively.

The DSC heating and cooling curves (Fig. 3a) show peaks at 53.5 °C and 48.3 °C, respectively, which correspond to the phase transition from tetragonal system (low temperature, antiferroelectric phase) to cubic system (high temperature, paraelectric phase). The T_c_ = 51 °C can be calculated from the DSC result. We also observed that the piezoelectric switching of ***a*****′**-axis to ***c*****′**-axis (spontaneous polarization direction) can be achieved by applying a mechanical pressure (Fig. S1). The CH_3_NH_3_PbI_3−x_Cl_x_ perovskite shows a maximum dielectric constant around T_c_ (51 °C), similar to the most ferroelectric and antiferroelectric metal oxide perovskites (Fig. 3b). Such dielectric behavior is a character of ferroelectric and antiferroelectric materials.

The results of neutron diffraction studies have suggested the possibility of formation of either antiferroelectric or ferroelectric domains in CH_3_NH_3_PbI_3_ perovskite with reducing the temperature based on the ordered orientation of CH_3_NH_3_^+^ cation^17^,^18^,^19^. However, the highly disordered orientation of CH_3_NH_3_^+^ cation around room temperature excludes possibility of the spontaneous polarization in the antiferroelectrics or ferroelectrics around room temperature owing to the ordered orientation of CH_3_NH_3_^+^ cation. Except ordered orientation of CH_3_NH_3_^+^ cation, the spontaneous polarization is also possible due to the displacement of positive and negative charge centroids generated by shifting the position of Pb(II) ion in the PbI_6_ octahedron along the c′ axis as well as the displacement of CH_3_NH_3_^+^ cation position in the organic-inorganic perovskites similar to the most ferroelectric and antiferroelectric metal oxide perovskites^20^,^21^,^25^. In the present study, our results confirm CH_3_NH_3_PbI_3−x_Cl_x_ perovskite is an antiferroelectric material and it can be switched to the ferroelectric phase after poling. The structural studies on the organic-inorganic perovskites have revealed straightening of the Pb–I–Pb bonds with increasing temperature from the tetragonal phase to cubic phase and the distortions of PbI_6_ octahedra from ideal even for the cubic lattice^19^. To further understand the antiferroelectric mechanism of CH_3_NH_3_PbI_3−x_Cl_x_ perovskite, a detail structural analysis is necessary, and identifying the origin of the antiferroelectricity would be a challenging study in the future.

### Ferroelectric-semiconductor solar cell

**Figure 4:**
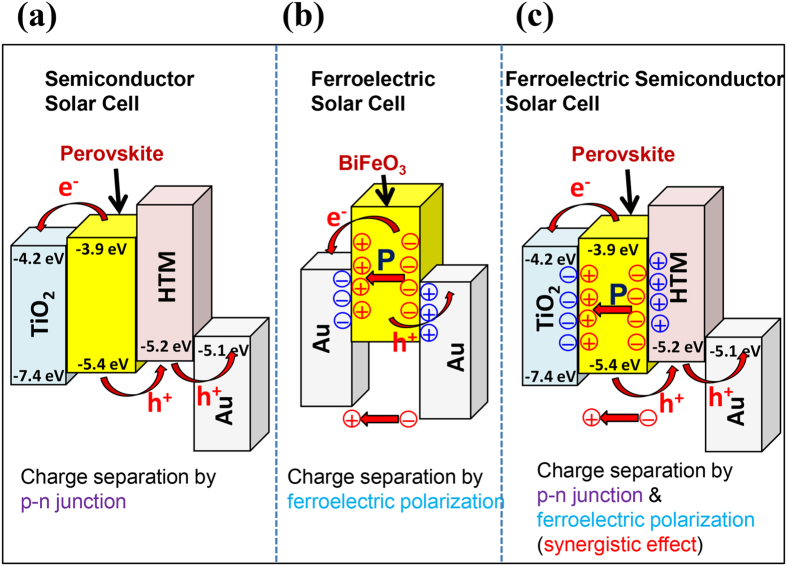
Charge separation mechanisms in (**a**) semiconductor solar cells, (**b**) ferroelectric solar cells, and (**c**) ferroelectric semiconductor solar cells.

We think solar cells can be categorized as conventional semiconductor p-n junction, ferroelectric, and new-type ferroelectric-semiconducting solar cells (Fig. 4). A synergistic effect of ferroelectric and semiconducting nature in the new-type ferroelectric-semiconducting solar cells can be clearly elucidated by using the charge separation mechanisms of semiconductor solar cells and ferroelectric solar cells. In the conventional semiconductor (p-n junction) solar cell (Fig. 4a), a semiconducting material absorbs photons with energies above the band gap and promotes electrons from the valence band to the conduction band. The generated hole and electron carriers are separated to the contacts due to the potential difference at the interfaces (perovskite/TiO_2_ and perovskite/HTM). In the ferroelectric solar cell (Fig. 4b), a poled ferroelectric material such as BiFeO_3_ separates photo-generated electrons and holes into contacts by the ferroelectric polarization; where, anode and cathode use the same material, such as Au^31^. The ferroelectric carrier separation effect has been observed in ferroelectric metal oxides and organic materials after poling treatment^31^,^32^.

Figure 4c represents the effective charge separation by the synergistic effect of ferroelectric and semiconducting behavior of CH_3_NH_3_PbI_3−x_Cl_x_ perovskite. Firstly, an initial photo-induced internal-electric field is generated by the p-n junction similar to the conventional semiconductor solar cells (Fig. 4a). Then the perovskite absorber layer is poled by the initial photo-induced internal-electric field, and the poled perovskite layer promotes the charge separation similar to the ferroelectric solar cell (Fig. 4b). Therefore, the synergistic effect of semiconducting and ferroelectric charge separations can be obtained in the ferroelectric CH_3_NH_3_PbI_3−x_Cl_x_ perovskite solar cells. Because the initial photo-induced internal-electric field is generated by the semiconducting behavior of the perovskite absorber layer (the self-poling effect), the poling treatment is not necessary for ferroelectric semiconductor solar cells, which is different from the conventional ferroelectric solar cells. The ideal photovoltaic material should separate charges as efficiently as possible and transport them independently to the contacts, to minimize recombination between electrons and holes^33^. Therefore, CH_3_NH_3_PbI_3−x_Cl_x_ perovskite is an ideal photovoltaic material due to its ferroelectric and semiconducting nature.

## Conclusion

Ferroelectric hysteresis loops of CH_3_NH_3_PbI_3−x_Cl_x_ perovskite were analyzed. The CH_3_NH_3_PbI_3−x_Cl_x_ perovskite is an antiferroelectric semiconductor different from the typical semiconducting and ferroelectric materials. The CH_3_NH_3_PbI_3−x_Cl_x_ perovskite exhibits ferroelectric-like P-E hysteresis loops and dielectric behavior around room temperature. The antiferroelectric CH_3_NH_3_PbI_3−x_Cl_x_ based PSCs, under working condition, can generate about 1.2 μC/cm^2^ polarization and it can promote the charge separation. A clear understanding of the perovskite absorber material is imperative for future developments in the ferroelectric semiconductor PSCs and the manipulation of ferroelectrics of the absorber materials is a promising strategy to optimize the cell performances.

## Additional Information

**How to cite this article**: Sewvandi, G. A. *et al*. Antiferroelectric Nature of CH_3_NH_3_PbI_3−x_Cl_x_ Perovskite and Its Implication for Charge Separation in Perovskite Solar Cells. *Sci. Rep.* **6**, 30680; doi: 10.1038/srep30680 (2016).

## Supplementary Material

Supplementary Information
