# Supplementary material for: Antiferroelectric Nature of CH3NH3PbI3−xClx Perovskite and Its Implication for Charge Separation in Perovskite Solar Cells
Source: Sci Rep. 2016 Jul 29;6:30680. doi: 10.1038/srep30680 (PMC4965787; doi:10.1038/srep30680)
Supplement: Supplementary Information [file srep30680-s1.pdf]

Electronic Supplementary Information

Antiferroelectric Nature of $CH_3NH_3PbI_{3-x}Cl_x$ Perovskite and Its Implication for Charge Separation in Perovskite Solar Cells

Galhenage A. Sewvandi [1,2] *Kei Kodera,* [2] *Hao Ma,* [2] *Shunsuke Nakanishi,* [2] *and Qi Feng* [2]*

[1]Department of Materials Science and Engineering, Faculty of Engineering, University of Moratuwa, Katubedda, Sri Lanka

[2]Department of Advanced Materials Science, Faculty of Engineering, Kagawa University, 2217-20 Hayashi-cho, Takamatsu 761-0396, Japan

Corresponding Author: *E-mail: feng@eng.kagawa-u.ac.jp.

Synthesis and Characterization

Chemicals and reagents. Hydroiodic acid (57% in water), and PbI_2 (98%) were purchased from Sigma-Aldrich. Methylamine (40% in methanol), $PbCl_2$ (99%), γ-butyrolactone (GBL), and N, N-dimethylformamide (DMF) were bought from Wako. All chemicals and reagents were analytical grade and used as received.

Synthesis of $CH_3NH_3PbI_{3-x}Cl_x$ powder. CH_3NH_3I was synthesized by reacting 24 mL of methylamine and 10 mL of hydroiodic acid at 0 °C for 2 h in a rotary evaporator at 120 rpm. The solution was evaporated at 80 °C and the precipitate was washed three times in ethanol followed by diethyl either at room temperature for 30 min. The collected precipitate was dried at 60°C for 24 h. To obtain $CH_3NH_3PbI_{3-x}Cl_x$ powder, the synthesized CH_3NH_3I powder was mixed with $PbCl_2$ at a 3:1 mole ratio in DMF:GBL= 9:1 mixed solvent at room temperature and the solution was dried on a glass plate at 150 °C.

Fabrication of $CH_3NH_3PbI_{3-x}Cl_x$ pellets. $CH_3NH_3PbI_{3-x}Cl_x$ pellets were fabricated for ferroelectric measurements. Synthesized $CH_3NH_3PbI_{3-x}Cl_x$ powder was gently ground into fine powder. This powder was then pressed at room temperature and 30 MPa pressure to produce pellets with a diameter of 10 mm and the thickness in a range of 500-600 µm.

Characterization. The structural characteristics and purity of the synthesized powders were investigated using SHIMADZU XRD-6100 X-ray diffractometer with a Cu Kα (λ = 0.15418) X-ray tube operated at 40 KV and 30 mA using a step size of 0.004° and a scan speed of 0.1°/min. Differential scanning calorimetry (DSC) was conducted on a NETZSCH DSC 200 F3 at a rate of 10 ºC min^{-1} over a temperature range from 0 °C to 150 ºC under nitrogen. Polarization-electric field (P-E) hysteresis loops of the $CH_3NH_3PbI_{3-x}Cl_x$ pellet samples at room temperature were obtained by using a standard method for the ferroelectric measurements on a TOYO Corporation FCE3-4KVSY ferroelectric testing system. The P-E hysteresis loops measurements were carried out at 2 kHz. The conductivity of the pellet sample was estimated from its leakage current.

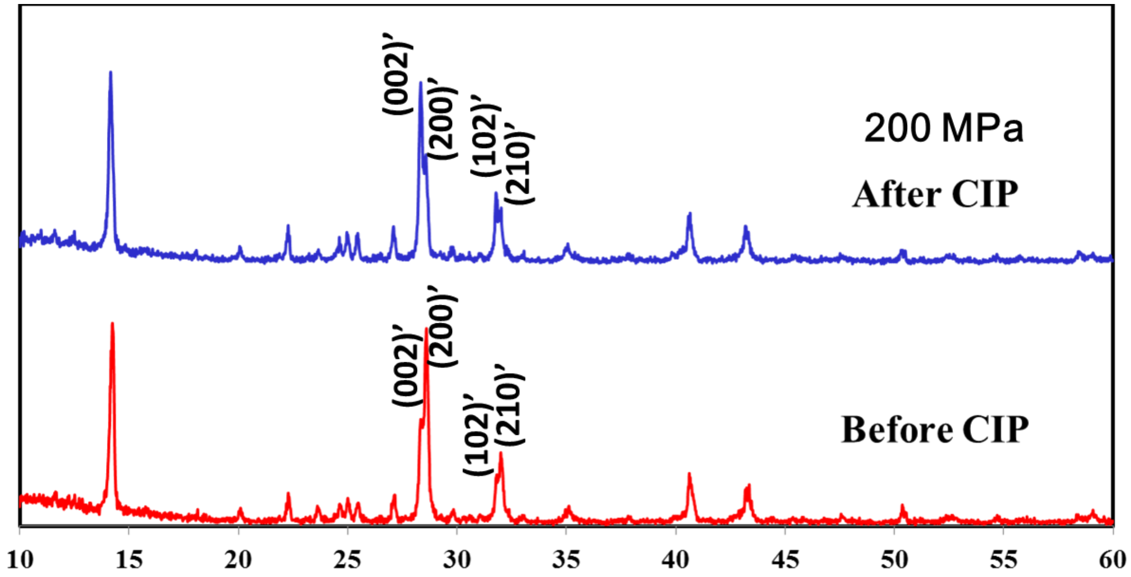

Fig. S1. XRD patterns of $CH_3NH_3PbI_{3-x}Cl_x$ perovskite pellet sample before and after CIP (cold isostatic press) treatment at 200 mPa pressure for 10 min.

The peak intensity ratio of (200)' / (002)' of the pellet sample of $CH_3NH_3PbI_{3-x}Cl_x$ perovskite is 2/1 before the CIP treatment same as the powder sample. However, the peak intensity ratio of (200)' / (002)' changed to 1/2 after the CIP treatment. This result indicates that the $CH_3NH_3PbI_{3-x}Cl_x$ perovskite pellet sample changed from a random orientation to [002]' orientation after the CIP treatment, suggesting a'-axis is transformed to c'-axis along mechanical force applied direction and gives a crystal-axis orientation along mechanical force applied direction. This behavior corresponds to the piezoelectric effect.
